# Chitin–glucan and pomegranate polyphenols improve endothelial dysfunction

**DOI:** 10.1038/s41598-019-50700-4

**Published:** 2019-10-02

**Authors:** Audrey M. Neyrinck, Emilie Catry, Bernard Taminiau, Patrice D. Cani, Laure B. Bindels, Georges Daube, Chantal Dessy, Nathalie M. Delzenne

**Affiliations:** 1 Metabolism and Nutrition research group, Louvain Drug Research Institute, UCLouvain, Université catholique de Louvain, Brussels, Belgium; 2 Fundamental and Applied Research for Animal and Health (FARAH), Faculty of Veterinary Medicine, University of Liège, Liège, Belgium; 3 Pole of Pharmacology and Therapeutics, Institut de Recherche Expérimentale et Clinique, UCLouvain, Université catholique de Louvain, Brussels, Belgium; 4 Walloon Excellence in Life Sciences and Biotechnology (WELBIO), UCLouvain, Catholic University of Louvain for Université catholique de Louvain, Brussels, Belgium

**Keywords:** Physiology, Risk factors

## Abstract

The vascular dysfunction is the primary event in the occurrence of cardio-vascular risk, and no treatment exists until now. We tested for the first time the hypothesis that chitin-glucan (CG) - an insoluble fibre with prebiotic properties- and polyphenol-rich pomegranate peel extract (PPE) can improve endothelial and inflammatory disorders in a mouse model of cardiovascular disease (CVD), namely by modulating the gut microbiota. Male Apolipoprotein E knock-out (ApoE−/−) mice fed a high fat (HF) diet developed a significant endothelial dysfunction attested by atherosclerotic plaques and increasing abundance of caveolin-1 in aorta. The supplementation with CG + PPE in the HF diet reduced inflammatory markers both in the liver and in the visceral adipose tissue together with a reduction of hepatic triglycerides. In addition, it increased the activating form of endothelial NO-synthase in mesenteric arteries and the heme-nitrosylated haemoglobin (Hb-NO) blood levels as compared with HF fed ApoE−/− mice, suggesting a higher capacity of mesenteric arteries to produce nitric oxide (NO). This study allows to pinpoint gut bacteria, namely *Lactobacillus* and *Alistipes*, that could be implicated in the management of endothelial and inflammatory dysfunctions associated with CVD, and to unravel the role of nutrition in the modulation of those bacteria.

## Introduction

Despite the enormous progress made in the diagnosis and treatment of cardiovascular diseases (CVDs) over the last twenty years, CVDs remain the leading cause of disability and death in Western countries^1^. Given the growing prevalence of obesity, type 2 diabetes and metabolic syndrome, it is estimated that the number of people with chronic cardiometabolic disease will continue to grow in the next two decades^1,2^. Obese individuals that develop atherosclerotic CVDs are often characterized by a low-grade chronic inflammation. It has been hypothesized that changes in inflammatory status partly explain the individual differences in cardiovascular risk^2^. Endothelial dysfunction is an early key marker of CVDs reflecting the integrated effects of risk factors on the vascular system^3^. It comes from the incapacity of endothelial cells to equilibrate synthesis and release of damaging or protective mediators, among which nitric oxide (NO) is the most important. The main feature of endothelial dysfunction is the inability of endothelium to promote vasodilation in response to agonist or shear forces^4^.

Apolipoprotein E (ApoE) is a key mediator for transport and reuptake of cholesterol. ApoE knock-out mice (ApoE−/−) are proposed as a model of atherosclerosis and endothelial dysfunction^5^. Endothelium-dependent relaxation is reduced in response to acetylcholine in 13-months-old ApoE−/− mice compared to wild-type mice^6^. However, this is not observed in youngest mice, suggesting that vascular disorders occurring in ApoE deficiency depend on age. Obesity are associated to altered vascular functions, with impaired vessel reactivity and lower endothelium-dependent relaxation. Interestingly, high fat (HF) diet feeding accelerates the process of atherosclerosis in ApoE−/− mice^7^. In addition, HF diets participate to worsening metabolic and vascular phenotypes which negatively influences the evolution of CVD in this murine model^8,9^.

The pathophysiology of CVDs is complex and involves multiple organs, establishing distant communications via bloodstream. Recently, a growing body of evidence has emerged regarding the critical role of gut microbiota in CVDs and metabolic disorders^1,10^. The gut microbiota can be considered as a “microbial organ” under the influence of the diet and host factors. Hence, the gut microbiota and its activity are intricately intertwined with the physiology and pathophysiology of the host^11–13^. Obesity in humans has been associated with the composition and function of the gut microbiota, but only a few studies focus on the role of gut microbiota in the CVDs^1^.

Modulation of the diet with specific dietary components could be used to prevent atherosclerosis. Dietary fibre is thought to have beneficial effects on health because it is considered to reduce the risk of CVD. In humans, this effect could be attributed to a decrease in plasma LDL^10,14^. For years, we and others have demonstrated that dietary fibre supplementation modulates the composition of the gut microbiota, and eventually improves host physiology^13,15,16^. This is particularly the case for inulin-type fructans (ITF), which are classified as prebiotics. Prebiotics are ‘substrates that are selectively utilized by host microorganisms conferring a health benefit’^17^. Whether CVD and atherosclerosis can be successfully treated by fibre targeting the microbiota is still not clear. ITF-fed ApoE −/− mice have been shown to have reduced cholesterol and atherosclerotic lesions compared to control mice. However, the mechanism was unexplored^18^. Recently, we demonstrated that ITF improve endothelial dysfunction in ApoE−/− mice fed a n-3 PUFA-depleted diet and developing steatosis. We found that ITF-induced changes in gut microbiota linked endogenous GLP-1 production with an improved endothelial function^19^. If proven in humans, those studies suggest that prebiotics might be proposed as a novel approach in the prevention of metabolic disorders-related CVDs.

Chitin-glucan (CG) has been shown to modulate the human gut microbiota in the *in vitro* SHIME microbiota simulator^20^. In a previous study, we have highlighted beneficial effects of this insoluble fibre on the development of obesity and associated diabetes and hepatic steatosis in mice, through a mechanism related to the restoration of the composition and/or the activity of gut bacteria^21^.

In the present study, we have tested the prebiotic potency of chitin-glucan, an insoluble dietary fibre, alone or in combination with a pomegranate peel extract (PPE) rich in polyphenols in a model of accelerated atherosclerosis in ApoE−/− mice fed a high fat diet during 8 weeks.

## Results

### Chitin-glucan supplementation with or without pomegranate peel extract did not change high fat diet-induced body weight gain, fat mass expansion and hypercholesterolemia

**Figure 1:**
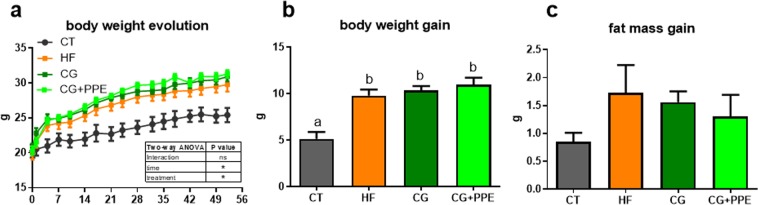
Body weight evolution (**a**), body weight gain (**b**) and fat mass gain (**c**) of ApoE−/− mice fed a low fat diet (CT), a high fat (HF) diet or a HF diet supplemented with 5% chitin-glucan (CG) or a combination of 5% CG and 0.5% pomegranate peel extracts (CG + PPE) for 8 weeks. Data with different superscript letters are significantly different at p < 0.05 (one-way ANOVA); ns = non significant and *p < 0.05 (two-way ANOVA).

The HF diet significantly increased body weight gain and the development of epididymal, visceral and subcutaneous adipose tissues of ApoE−/− mice as compared with control diet (Fig. 1 and Table 1). The fat mass increased upon HF feeding without reaching significance (Fig. 1). Liver weight and cholesterolemia were higher in HF-fed than in CT mice (Table 1). CG or CG + PPE supplementation did not significantly change those parameters. The lipid profile in the plasma was not affected by the dietary treatments (Table 1). Although this effect was not statistically significant due to large variability, CG with or without PPE decreased the level of ALAT in the serum (ALAT activity expressed in U/L: 16.4 ± 6.6, 16.6 ± 5.2, 6.7 ± 1.2, 8.8 ± 1.9 for CT, HF, CG and CG + PPE groups, respectively).

**Table 1:** Organ weights and plasma lipids.

	CT	HF	CG	CG + PPE
Liver (g)	0.96 ± 0.08^a^	1.21 ± 0.07^b^	1.25 ± 0.06^b^	1.24 ± 0.03^b^
Visceral adipose tissue (%)	0.44 ± 0.05	0.65 ± 0.07	0.59 ± 0.07	0.55 ± 0.07
Subcutaneous adipose tissue (%)	0.80 ± 0.06^a^	1.45 ± 0.20^b^	1.19 ± 0.11^ab^	1.14 ± 0.14^ab^
Epididymal adipose tissue (%)	0.96 ± 0.08^a^	1.88 ± 0.27^b^	1.54 ± 0.17^b^	1.51 ± 0.17^ab^
Cecal tissue (mg)	56 ± 5^a^	41 ± 2^b^	51 ± 2^ab^	56 ± 2^a^
Cecal content (mg)	239 ± 27	171 ± 15	221 ± 11	213 ± 17
Triglycerides (mM)	0.71 ± 0.09	0.73 ± 0.17	0.59 ± 0.06	0.60 ± 0.07
Non esterified fatty acids (mM)	0.54 ± 0.02	0.56 ± 0.09	0.45 ± 0.06	0.48 ± 0.06
Total cholesterol (mM)	9.78 ± 1.55^a^	16.68 ± 2.24^b^	14.92 ± 0.76^b^	15.90 ± 0.73^b^

ApoE−/− mice were fed a low fat diet (CT), a high fat (HF) diet or a HF diet supplemented with 5% chitin-glucan (CG) or a combination of 5% CG and 0.5% pomegranate peel extracts (CG + PPE) for 8 weeks. Data with different superscript letters are significantly different at p < 0.05 (ANOVA).

It is interesting to note that ApoE−/− mice upon HF feeding and treated with CG or CG + PPE exhibited the higher cecal tissue and cecal content weight compared to HF group; this effect reached statistical significance for the cecal tissue from the mice treated with the combination (Table 1).

### Chitin-glucan supplementation with pomegranate peel extract decreased hepatic content of triglycerides

**Figure 2:**
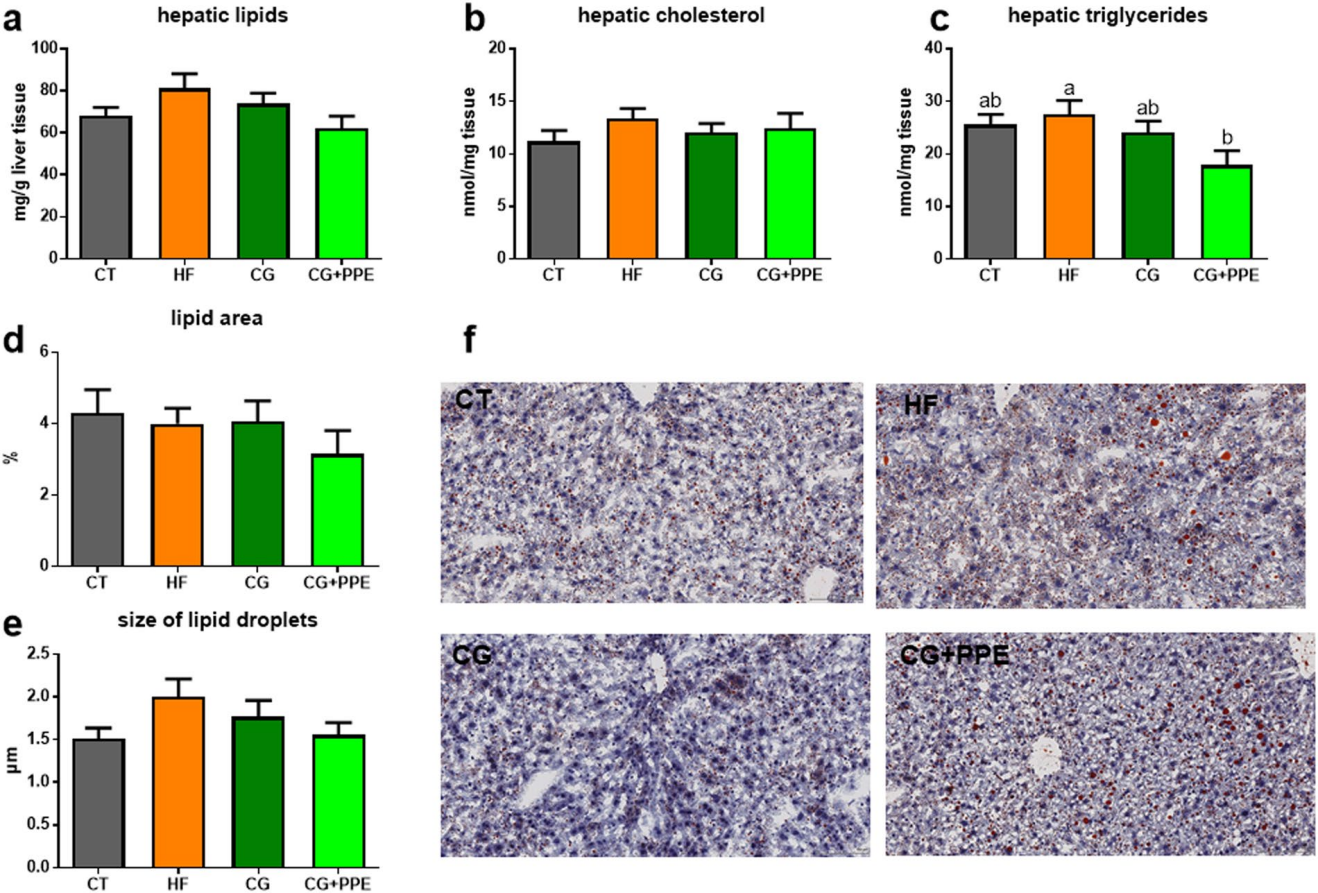
Lipid accumulation in the liver tissue. Hepatic content of lipids (**a**), cholesterol (**b**) and triglycerides (**c**) of ApoE−/− mice fed a low fat diet (CT), a high fat (HF) diet or a HF diet supplemented with 5% chitin-glucan (CG) or a combination of 5% CG and 0.5% pomegranate peel extracts (CG + PPE) for 8 weeks. Lipid fraction area (**d)** and mean size of lipid droplets (**e**) were automatically analysed by ImageJ from Oil red O staining of frozen section of the main lobe of liver, bar = 100 µm (**f**). Data with different superscript letters are significantly different at p < 0.05 (ANOVA).

The HF diet did not significantly affect lipid content, cholesterol content and triglyceride content of the liver tissue from the ApoE−/− mice compared to CT group as shown in Fig. 2(a–c). The supplementation with the CG in combination with PPE significantly reduced hepatic triglycerides (Fig. 2c). Fat staining of the tissue confirmed the lower fat accumulation in the liver even if the significance was not reached (Fig. 2d–f).

In addition, we measured the expression of two hepatic genes regulating lipid metabolism: fatty acid synthase (*Fas*), a key enzyme involved in lipogenesis and carnitine palmitoyl transferase-1 (*Cpt1*), a marker of fatty acid oxidation. None of the dietary treatments significantly affect *Fas* or *Cpt1* expression (data not shown).

### Chitin-glucan supplementation with pomegranate peel extract reduced inflammatory markers both in adipose tissue and in the liver

**Figure 3:**
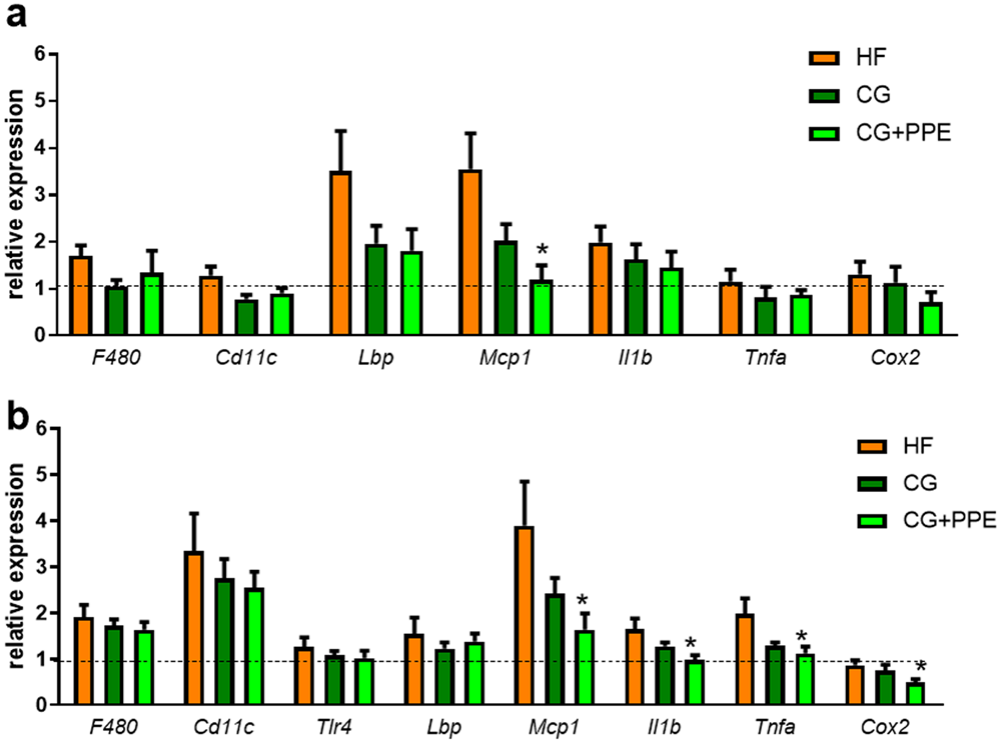
Expression of inflammatory markers in the visceral adipose tissue (**a**) and the liver (**b**). ApoE−/− mice were fed a high fat (HF) diet or a HF diet supplemented with 5% chitin-glucan (CG) or a combination of 5% CG and 0.5% pomegranate peel extracts (CG + PPE) for 8 weeks. Data are expressed as the mean ± SEM. The dotted line depicts the relative values observed in ApoE−/− mice fed a low fat diet (set at 1). *p < 0.05 versus HF group (ANOVA).

HF diet has been reported to induce endotoxemia and inflammation in the liver and the visceral adipose tissue^22^. In addition, our previous findings support that pomegranate extract alleviated tissue inflammation in HF diet-induced obese mice^23^. Therefore, we analyzed the expression of the target genes among others, in particular: two macrophage markers (CD11c, F4/80), the lipopolysaccharide binding protein (LBP), a key pattern recognition receptor (Toll-like Receptor-4 (TLR4)), one of the most potent chemokines identified for monocytes recruitment (monocyte chemotactic protein-1 (MCP1)), two important proinflammatory cytokines (tumor necrosis factor alpha (TNFα) and interleukin-1β (Il1β)) and the gene coding for cyclooxygenase (COX-2) that produces prostaglandin E2, a regulator of inflammation, both in the liver and the visceral adipose tissues^24^. Several markers of macrophage infiltration and/or inflammation were induced in the visceral adipose tissue (p < 0.05 ANOVA for *Mcp1*) and in the liver (p < 0.05 ANOVA for *Mcp1, Cd11c, Il1b, Tnfa, F480*) of ApoE−/− mice due to HF feeding as compared to CT group (Fig. 3). Among them, MCP1 was downregulated after CG supplementation; this effect was more pronounced and significant in combination with the PPE. In addition, the expression of the proinflammatory cytokine TNFα, Ilβ and COX-2 were downregulated in the liver from mice treated with the combined supplementation (Fig. 3b). However, several circulating cytokines and biomarkers of inflammatory related to cell adhesion molecules (IL-6, IL-10, IL-1β, MIP1α, MCP1, TNFα, sE-Selectin, sICAM-1, PAI-1, proMMP-9) were not significantly influenced by the treatments (data not shown).

### Chitin-glucan supplementation with pomegranate peel extract improved endothelial (dys)function

**Figure 4:**
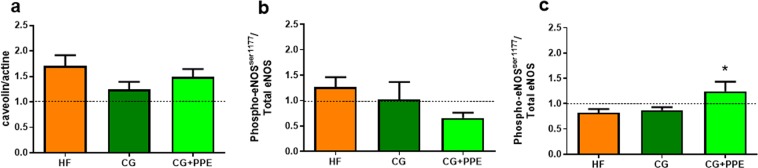
Western blot analyses on aorta (**a,b**) and mesenteric arteries (**c**) with anti-caveolin-1 (**a**) or anti-phosphorylated endothelial NOS (eNOS)^ser1177^ (**b**,**c**). ApoE−/− mice were fed a high fat (HF) diet or a HF diet supplemented with 5% chitin-glucan (CG) or a combination of 5% CG and 0.5% pomegranate peel extracts (CG + PPE) for 8 weeks. Data are expressed as the mean ± SEM. Values are expressed relative to ApoE−/− mice fed a low fat diet (set at 1). *p < 0.05 versus HF group (ANOVA).

Endothelial dysfunction was attested by atherosclerotic plaques in aorta isolated from ApoE^−/−^ mice fed a HF diet (data not shown). In line with a potential reduced endothelial function^25^, aorta showed increased abundance of caveolin-1 (*p < 0.05 HF group versus CT group, ANOVA), the negative allosteric regulator of endothelial NO synthase (eNOS) (Fig. 4a). We performed western blotting to assess the activating serine 1177 phosphorylated form of endothelial NOS (p-eNOS^ser1177^) in conductance (thoracic) versus resistance (mesenteric) arteries (Fig. 4b,c). Interestingly, the combination CG + PPE increased the activating form of eNOS in mesenteric arteries (Fig. 4c).

**Figure 5:**
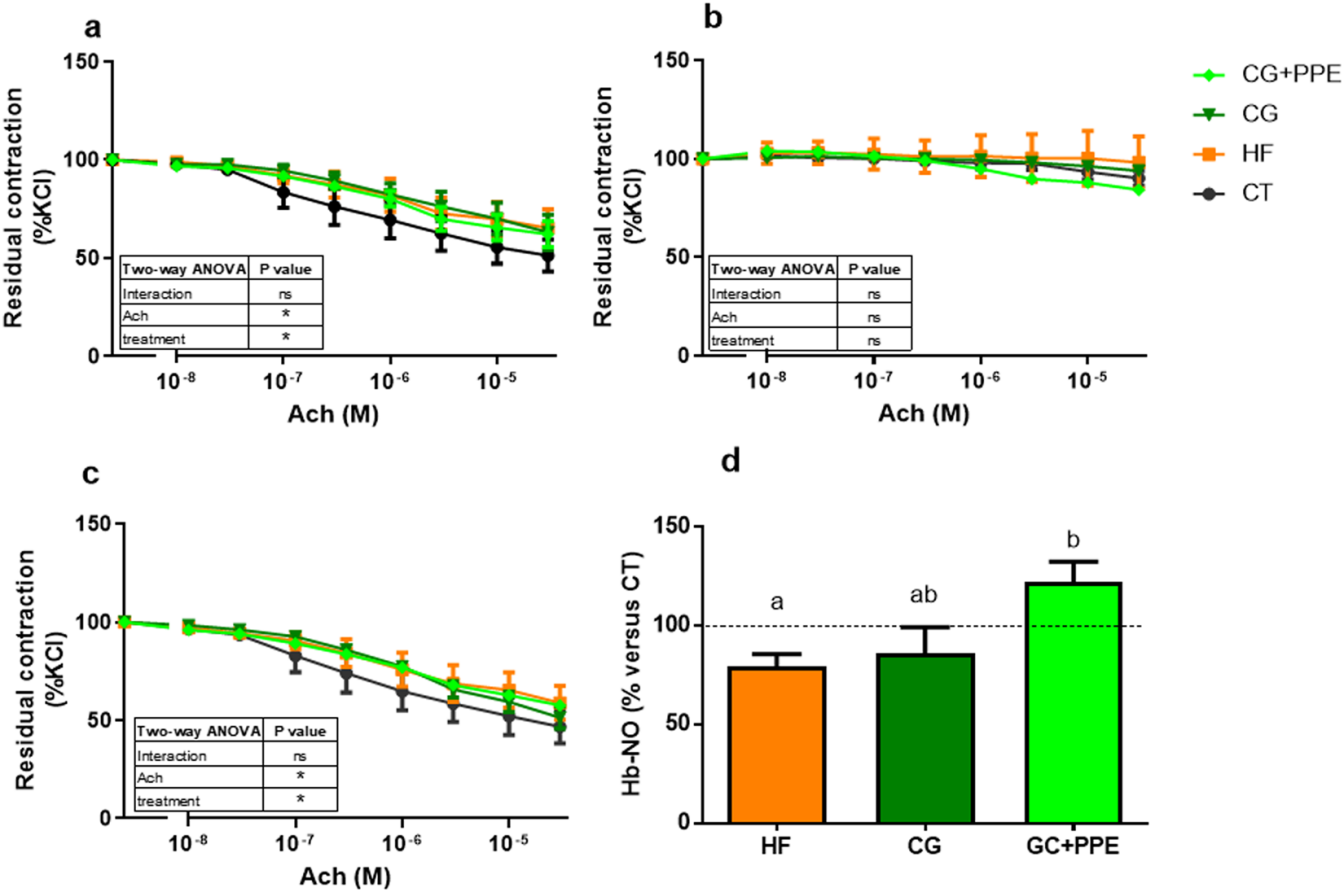
Endothelium-dependent relaxation of preconstricted mesenteric arteries and level of Hb-NO in venous blood. Endothelium-dependent relaxation was evaluated by cumulative addition of acetylcholine (Ach) on pre-contracted arteries with a high KCl-solution, in the absence (**a**) or the presence of nitric oxide synthase inhibitor Nω-Nitro-L-arginine methyl ester (**b**) or a cyclooxygenase inhibitor (**c**). Level of Hb-NO in venous blood (**d**). ApoE−/− mice were fed a low fat diet (CT), a high fat (HF) diet or a HF diet supplemented with 5% chitin-glucan (CG) or a combination of 5% CG and 0.5% pomegranate peel extracts (CG + PPE) for 8 weeks. Data with different superscript letters are significantly different at p < 0.05 (one-way ANOVA); ns = non significant and *p < 0.05 (two-way ANOVA).

Mesenteric arteries segments were mounted in wire myograph in order to evaluate endothelial function. Resting parameters revealed no difference (after normalization) between the four groups concerning the basal tone, the maximal contraction after a KCl challenge and the mean arterial diameter (supplemental File 1). The endothelium-dependent relaxation was studied upon acetylcholine addition in the incubation medium on pre-constricted arteries in the presence of a KCl-enriched solution and in the presence of a cyclooxygenase inhibitor (indomethacin) or a NO synthase inhibitor (L-NAME). Mesenteric arteries isolated from HF treated ApoE−/− mice relaxed less in response to acetylcholine than vessels obtained from ApoE−/− mice fed a control diet, suggesting the presence of endothelial dysfunction in HF fed mice whatever the supplementation (Fig. 5a). In contrast to incubation in the presence of indomethacin, this effect disappeared in the presence of NO synthase inhibitor (Fig. 5b), suggesting that HF diet worsened the NO-dependent endothelial dysfunction of ApoE−/− in mesenteric arteries. However, dietary supplementations did not significantly modify endothelium-dependent relaxation of mesenteric arteries (Fig. 5a–c). Importantly, to confirm an impact of CG + PPE on NOS/NO pathway, we measured the level of circulating heme-nitrosylated hemoglobin (Hb-NO) in the mice venous blood by EPR (Fig. 5d). HF mice presented a decreased level of Hb-NO compared to CT mice. In line with result obtained with phosphorylated form of eNOS protein, CG + PPE mice displayed a 40% increase in Hb-NO levels as compared with HF mice.

### Chitin-glucan supplementation with pomegranate peel extract affected the gut microbiota composition in ApoE−/− mice fed a high fat diet

**Figure 6:**
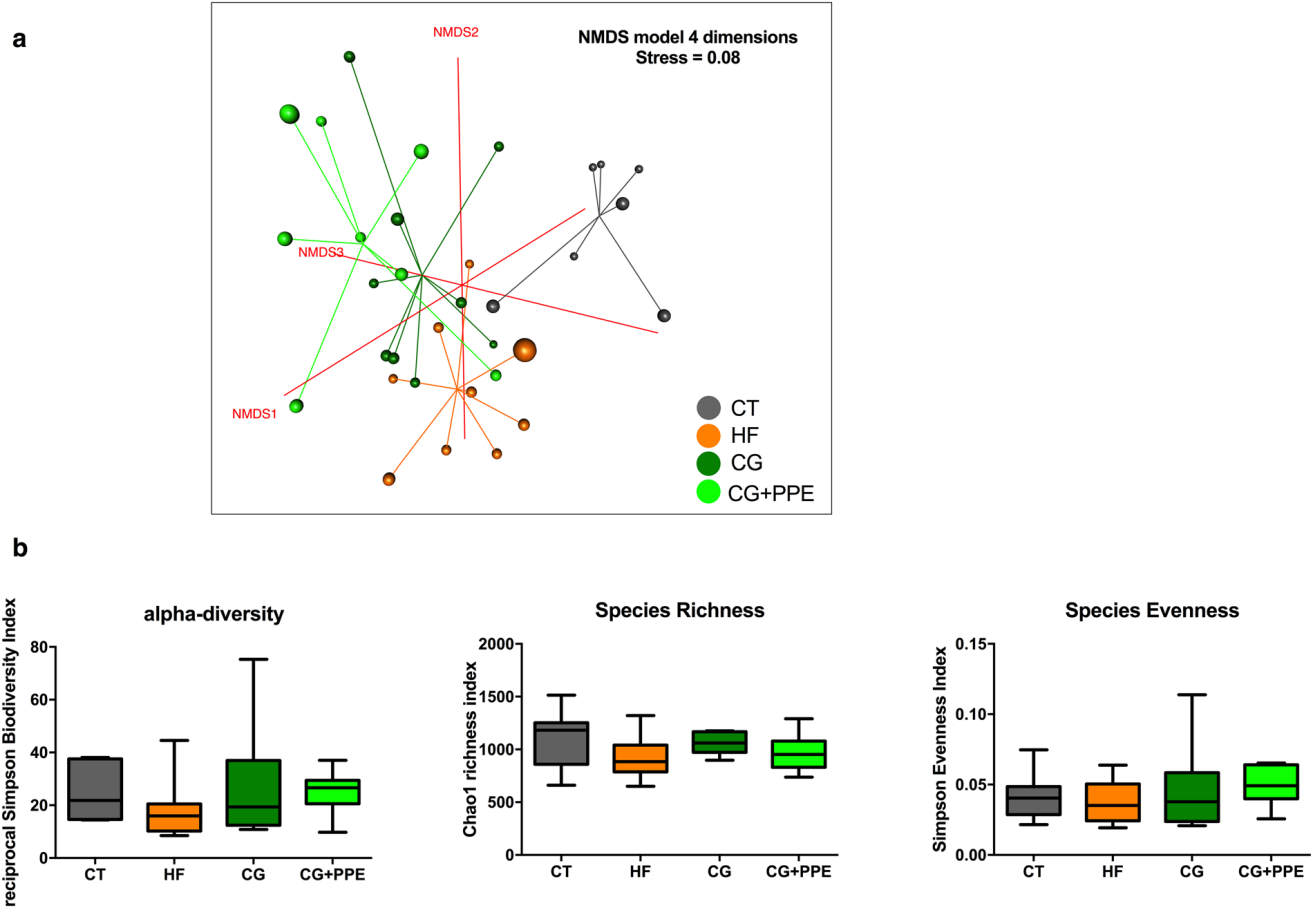
Microbiota structure assessed by non-metric dimensional scaling (NMDS) and bacterial diversity. A four dimensional model, built on a species level dissimilarity matrix has been obtained with a stress value of 0.08 (**a**). Reciprocal Simpson alpha-diversity, Chao richness and Simpson evenness (**b**) assessed from amplicon sequencing data of microbial cecal content of ApoE−/−mice fed, a low fat diet (CT), a high fat (HF) diet or a HF diet supplemented with 5% chitin-glucan (CG) or a combination of 5% CG and 0.5% pomegranate peel extracts (CG + PPE) for 8 weeks (p > 0.05 ANOVA, Kruskal-Wallis test).

**Figure 7:**
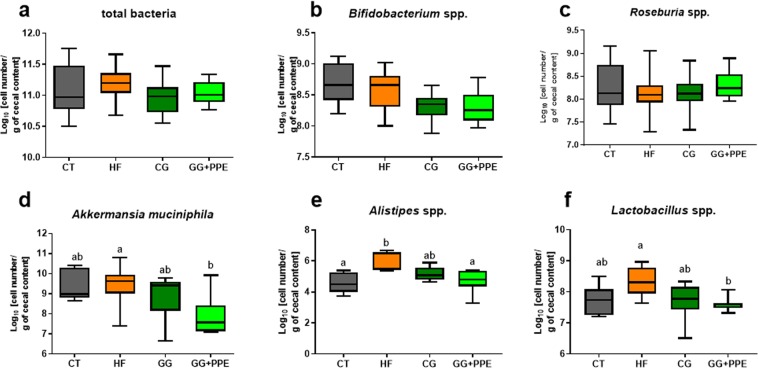
Cecal bacteria assessed by qPCR. Total bacteria (**a**), *Bifidobacterium* spp. (**b**), *Akkermansia muciniphila* (**c**), *Roseburia* spp. (**d**), *Alistipes spp.* (**e**), *Lactobacillus spp.* (**f**) in the cecal content of ApoE−/−mice fed, a low fat diet (CT), a high fat (HF) diet or a HF diet supplemented with 5% chitin-glucan (CG) or a combination of 5% CG and 0.5% pomegranate peel extracts (CG + PPE) for 8 weeks. Data are Whiskers plots with minimum and maximum. Data with different superscript letters are significantly different at p < 0.05 (ANOVA, Kruskal-Wallis test).

The differences within the intestinal microbial population between the four groups were visualized by Non Metric Dimensional Scaling built upon a Bray-curtis distance matrix based on the species taxonomic level (Fig. 6a). A distinct clustering was observed for each of the four groups of mice and was confirmed by AMOVA analysis of the dissimilarity matrix, albeit not for the CG + PPE versus CG groups (Supplementary File 1). We did not observe any significant effect on cecal microbial α-diversity (Fig. 6b). Despite the presence of different clustering, the relative abundances between phyla or family or genus due to CG and PPE supplementation were not significantly affected at the q-value by the dietary treatment (Supplementary Dataset 1). However, it is important to mention that the abundances of *Akkermansia* and *Alistipes* among other bacterial genera were significantly different between the dietary treatments at the p-value. In addition to the sequencing of the cecal microbiota, we analyzed some specific bacteria with a complementary quantitative approach by using qPCR method on an educated guess basis, meaning that we selected bacteria for which previous reports have shown a link between these bacteria and inflammation/metabolic disorders: (1) several studies demonstrated that specific *Bifidobacterium* or *Lactobacillus* strains alone or in combination, decreased the metabolic alterations (decrease of body weight and fat mass gain) together with a reduction of the inflammatory events, occurring in the liver and/or the adipose tissue in diet-induced obesity models^26,27^; (2) a recent paper demonstrated that *Roseburia* interacted with dietary plant polysaccharides to lower systemic inflammation and ameliorated atherosclerosis in germ-free apolipoprotein E-deficient mice colonized with synthetic microbial communities^28^. Moreover, our previous work showed that CG had metabolic interest that could be related to the increase in *Roseburia* spp. in a mouse model of HF induced-obesity^21^; (3) *Alistipes* were positively associated with HF-induced obesity in several independent studies^29–31^; (4) finally, *Akkermansia* is a bacterium capable of reducing adipose tissue inflammation and dyslipidaemia in obese mice^26,27,32,33^. The Fig. 7 shows that the increase of *Alistipes* spp. and *Lactobacillus* spp in the cecal content due to HF feeding was counteracted by the CG + PPE supplementation (Fig. 7e,f). *Akkermansia muciniphila* were also significantly decreased by the combination CG + PPE compared to the HF fed group (Fig. 7d). We did not observe any changes in total bacteria, *Roseburia* spp. and *Bifidobacterium* spp. (Fig. 7a–c).

## Discussion

The nutritional quality of diets underlies or exacerbates several chronic pathologies, including CVD^34,35^. Western diets are high in calories, simple sugars, saturated fat and are also characterized by an imbalance in the dietary intake of n-3 and n-6 PUFA^36^. In a recent study, we demonstrated that n-3 PUFA depletion for 12 weeks could accelerate the process of endothelium dysfunction in young ApoE−/− mice prone to develop atherosclerosis^19^. Another study demonstrated that consumption of HF diet (60% kJ from fat) during 17 weeks promotes obesity and accelerates atherosclerosis in ApoE−/− mice^7^. This effect was accompanied by the development of a metabolic syndrome phenotype characterized by a low level of inflammation and atherosclerotic lesion^7^. In the present study, we show that HF feeding (60% kcal from fat and 0.15% cholesterol) induces obesity, hypercholesterolemia, inflammatory disorders and endothelial dysfunction in young (10-wk-old at the beginning of the treatment) ApoE−/− mice fed a HF diet for only 8 weeks. We then used this model to test the health benefits of original nutrients in the management of endothelial and inflammatory dysfunctions associated with CVD.

The health-promoting effect of prebiotic fibre and plant extracts is subject to interesting developments^37^. Chitin-glucan (CG), a natural component of the cell wall of microscopic fungi has been approved as a novel food ingredient after a positive safety evaluation in 2010 by the European Food Safety Authority^38^. The CG used in the present study is a high-purity biopolymer extracted from the mycelium of *Aspergillus niger*. This insoluble fiber is composed of two types of polysaccharide chains, namely chitin (poly-N-acetyl-D-glucosamine) and beta-(1,3)-D-glucan (D-glucose units linked predominantly via beta-(1,3) linkage). We previously demonstrated its beneficial effects on the development of obesity and associated diabetes and hepatic steatosis in HF-fed mice, through a mechanism related to the restoration of the composition and/or the activity of gut bacteria [33]. On the other side, health benefits of pomegranate fruit and/or juice consumption have recently received considerable scientific focus. The PPE used in this study is a source of polyphenol (40%), with punicalagin and ellagic acid levels reaching 10% and 2%, respectively. Our previous findings support that pomegranate extract constitutes a promising food ingredient to control atherogenic and inflammatory disorders associated with diet-induced obesity since it alleviated tissue inflammation and hypercholesterolaemia in HF diet-induced obese mice^23^. In line with our previous results, here we observe that CG + PPE reduces several proinflammatory parameters such as the expression of the chemokine MCP-1 (involved in the recruitment of inflammatory cells inside the tissue) in both the visceral and liver tissues and the expression of the proinflammatory cytokines IL-1β and TNFα in the liver.

The presence of hepatic steatosis has previously been reported for ApoE−/− mice fed a HF diet and the model has been proposed to study features of nonalcoholic fatty liver disease (NAFLD) on cardiovascular events^38,39^. In the present study, HF feeding did not induce higher lipid accumulation in the liver of ApoE−/− mice than the low fat diet. This is probably due to the short-term dietary treatment investigated here. Importantly, the supplementation with CG + PPE reduced significantly hepatic triglycerides, one of the critical features of NAFLD. Further studies are needed to unravel the mechanism behind the lower hepatic fat accumulation since two key genes in the liver that regulate lipid metabolism (FAS and CPT-1) were not affected by the dietary treatments.

Metabolic diseases such as NAFLD are associated with macro- and microcirculation damage, initiating an impairment of endothelium-dependent relaxation^39,40^. Eight weeks of HF-diet were able to accelerate the process of endothelial dysfunction in aorta namely by increasing abundance of caveolin-1, a negative allosteric regulator of eNOS leading to endothelial dysfunction exacerbation and associated CVD^25,41^. However, the dietary combination did not affect this parameter in the aorta, the major representative conductance vessel of the macrocirculation. We used western blotting to assess the activation of eNOS through analysis of its phosphorylated forms (p-eNOS^ser1177^) both in conductance (thoracic) and resistance (mesenteric) arteries. We observed that the activated form of eNOS was a third higher in mesenteric arteries due to supplementation with CG + PPE compared to HF ApoE−/− mice, suggesting that the innovative dietary combination activated the NOS/NO pathway in the resistance arteries representing the microcirculation near the gut. Furthermore, we investigated the relaxation profile of mesenteric arteries. We demonstrated that HF feeding for 8 weeks accelerated the process of endothelial dysfunction in resistance mesenteric arteries in ApoE−/− genotype, as already shown in previous studies^42,43^. We concluded that prostanoids do not play an major role in this early stage of endothelial dysfunction, based on the lack of effect of indomethacin, a non-selective cyclooxygenase inhibitor. In most cases, endothelial dysfunction is related to a reduced NO availability in mice^6^. Accordingly, impairment of endothelial function due to HF feeding is mainly dependent on a reduced NO bioavailability in ApoE−/− mice as L-NAME (a NOS inhibitor), counteracted the vasorelaxation of mesenteric arteries from all mice and repealed the differences between genotypes, independently of the dietary conditions. In contrast, resting parameters (vessel diameter and basal tone) and contraction profile (response to KCl challenge stimulation) of mesenteric arteries were not affected by the HF treatment whatever the dietary supplementations. In our experimental setting, CG + PPE supplementation did not improve the NO-dependent endothelium relaxation measured in mesenteric arteries. Of more relevance for human CVD, the effects of CG + PPE supplementation were not restricted to the phosphorylated form of eNOS in enteric vascular tree since this dietary treatment also increased the circulating levels of Hb-NO in peripheral blood at distance from the mesenteric bed. Altogether, our results demonstrate that, besides interesting hepatic events highlighted above, the supplementation with CG and PPE for 8 wk improved the endothelial function with consequences on systemic NO bioavailability in HF-fed ApoE−/− mice. It was previously shown that pomegranate extract administrated alone during 30 days enhanced endothelium-dependent artery relaxation although it concerned coronary arteries isolated from perfused hearts of spontaneously hypertensive ovariectomized rats^44^. Furthermore, they demonstrated in the same study that the treatment with their extract inhibited p-eNOS Thr^495^ without affecting p-eNOS Ser^1177^, improved lipid profile and was able to prevent the reduction in plasma nitrite levels spontaneously induced by castration.

Gut microbiota plays a crucial role in the control of host intestinal functions and management of NAFLD, through the release and/or biotransformation of metabolites (eg, bile acids and short chain fatty acids) which regulate gut endocrine function^12^. Establishing the causal and consequential actions of the gut microbiota in driving obesity and related-metabolic diseases has been challenging. Interestingly, Stepankova *et al*.^45^ reported that germ-free ApoE−/− mice developed more aortic atherosclerotic plaques compared with conventionally raised ApoE−/− mice fed the same low standard cholesterol diet. Along the same lines, compared to germ-free mice, the levels of cholesterol and triglycerides in plasma of conventionally raised mice are reduced, whereas they are increased in adipose tissue and liver^46^. Both findings support the hypothesis of a protective ability for the gut microbiota in CVD and in the management of plasma levels of cholesterol and lipids^10^. We have previously shown that the improvement of vascular dysfunction by ITF was linked to an activation of the NOS/NO pathway, which could be dependent on events occurring at the gut microbiota level (namely, through an increase in NO-producing bacteria). Although dietary treatment induced clusterization due to CG and CG + PPE supplementation, our targeted microbiota characterization did not reveal any changes in bacterial diversity or relative abundance of bacteria whatever the taxonomical level considered. However, the CG and PPE counteracted the effect of the HF feeding on the abundance of *Alistipes* spp and *Lactobacillus* spp present in the cecal content of mice only when these compounds were combined. Recently, Moschen and colleagues identified *Alistipes* spp. as one of the top ten most abundant genera associated with human colorectal carcinoma and provide experimental evidence that *Alistipes* potently induces inflammation in colitis-prone Il10−/− mice^47^. In line with this finding, *Alistipes* were positively associated with HF-induced obesity in several independent studies^29–31^. The depletion of *Alistipes* by CG + PPE in the current study is particularly relevant, considering that in both studies, they demonstrated that transmissible microbial and metabolomic remodeling by dietary fiber improved metabolic homeostasis^29,30^. Our results indicated that *Lactobacillus* spp. and *Akkermansia muciniphila* are other bacteria susceptible to dietary manipulation. It has been reported that members of the *Lactobacillus, Akkermansia muciniphila* and *Bifidobacterium* genera may play a critical role as anti-obesity drivers in experimental models and humans^32,48,49^. One study linked specific species of *Lactobacillus* (*L. reuteri*) with obesity in humans^50^ whereas many studies tested for their ability to affect positively obesity and risk factors associated with CVD^26,51^. Here, the *Lactobacillus* genus increased upon HF feeding whereas the supplementation with CG + PPE blunted this effect. The study of Fak and Backhed demonstrated that bacterial strains of the same *Lactobacillus* species showed different effects on adiposity and insulin sensitivity in ApoE−/− mice, illustrating the complexity of host bacterial cross-talk and the importance of strain specificity^52^. In this context, it would be of interest to further characterize the identity and the functionality of the *Lactobacillus* strains affected by the dietary treatments. Although *Akkermansia muciniphila* is a bacterium capable of reducing fat mass development, insulin resistance, metabolic endotoxemia, adipose tissue inflammation and dyslipidaemia in obese mice and in a pilot study conducted in humans^32,33^, we observed a decreased abundance of this bacteria in the cecal content of ApoE−/− mice treated with the combination CG + PPE. Our previous work showed that CG had metabolic interest that could be dependent on the increase in *Roseburia* spp. in a mouse model of HF induced-obesity^21^ but their level was not impacted the dietary treatments in the model of HF-fed ApoE−/− mice. Therefore, we proposed that neither *Akkermansia* nor *Roseburia* spp. were involved in the metabolic and anti-inflammatory effects of the combined treatment (CG + PPE) observed in the present study. These data also clearly support the notion that we should not generalize the effects of dietary fibers and polyphenols on *Akkermansia*, as discussed in a recent review, predicting which fibers or polyphenols are the most suited to maximize beneficial health effects is highly dependent on the health situation, the individual and its microbiota^53^

In conclusion, our study demonstrated that food supplementation with CG and PPE limited triglyceride accumulation in the liver and exerted anti-inflammatory effects both in the liver and in the visceral adipose tissue of ApoE−/− mice fed a HF diet. In addition, the combination improved endothelial function in mesenteric arteries through a higher production and availability of NO in this model. Gut bacteria such as *Lactobacillus* and *Alistipes* could be implicated in the management of metabolic and inflammatory dysfunctions associated with CVD. The beneficial effects were observed only when CG was associated with PPE in the diet, confirming a previous study that highlighted the need to consider the interactions among bioactive food components when evaluating potential prebiotic effects of dietary fiber^54^.

## Methods

### Ethics statement

All experiments were performed in strict accordance with relevant guidelines and regulations for the care and use of animals and in accordance with the EU directive. All mouse experiments were approved by and performed in accordance with the guidelines of the local ethics committee for animal care of the Health Sector of the Université catholique de Louvain under the specific agreement numbers 2017/UCL/MD/005. Housing conditions were as specified by the Belgian Law of 29 May 2013, on the protection of laboratory animals (Agreement LA 1230314). Every effort was made to minimize animal pain, suffering, and distress and to reduce the number of animals used.

### Animals and experimental design

Nine weeks old male and ApoE−/− mice (B6.129P2-*Apoe*^*tm1Unc*^/J from Charles River Laboratories, L’Arbresle, France) were housed two or three mice per cage (environmental enrichment: plastic tunnels and sheets) and with a 12 h light/dark cycle at 22 °C, diet and water were ad libitum. Mice were fed a low fat (10 kJ%), no sucrose, purified ingredient diet (CT, E157452-047, Ssniff, Germany) or a high fat (HF) diet (60 kJ% fat, 0.15% cholesterol, S8899-E730, Ssniff, Germany) for 8 weeks. HF-fed mice were supplemented with or without 5% CG (Kitozyme, Belgium) and a combination of 5% CG and 0.5% PPE (Oxylent, Belgium). CG used in the study was derived from the cell walls of the mycelium of *Aspergillus niger*, in which two types of polysaccharide chains, i.e., chitin (poly N-acetyl-D-glucosamine) and β(1,3)-D-glucan, are associated. PPE extract (OXYLENT®GR, Oxylent S.A.) used in the study was derived from pomegranate peel in which polyphenol content reached 40% (Folin–Ciocalteu method, equivalent gallic acid) and the concentrations of punicalagin and ellagic acid were 10 and 2% (ultra-performance liquid chromatography method with diode array detection), respectively.

The data provided in the manuscript are issued from 2 separate experiments, performed in the same animal facility, one for measurement of vascular contraction and relaxation and western blot analysis of mesenteric arteries (experiment A, n = 8 per group) and one for other parameters (experiment B, n = 9 per group). Body composition was assessed every two weeks by using 7.5 MHz time domain-NMR (LF50 minispec, Bruker, Rheinstetten, Germany).

#### Experiment A

After a total of 8 weeks of dietary treatment, mice were anaesthetized in postprandial state (Anesketin®, Ketamin hydrochloride and Rompun®, xylazine hydrochloride i.p., 100 and 10 mg/kg of body weight, respectively). Second and third order mesenteric arteries were rapidly removed and carefully isolated from visceral adipose and/or connective tissues as previously described^55^.

#### Experiment B

After a total of 8 weeks of dietary treatment and a 6-h period of fasting, mice were anesthetized with isoflurane (Forene®, Abbott, Queenborough, Kent, England) and blood samples were harvested. We collected venous blood (0.2 mL) from the right ventricle, immediately frozen in heparinized calibrated tube in liquid nitrogen. Venous blood samples were also collected and centrifuged (3 min at 13,000 g) for further analysis. Liver, aorta, adipose tissues and caecum were carefully dissected, weighted and immersed in liquid nitrogen before storage at −80 °C; pieces of liver and aorta were embedded in OCT compound and frozen in nitrogen cooled isopentane for histology.

### Measurement of nitric oxide bioavailability by electron paramagnetic resonance

The level of circulating heme-nitrosylated haemoglobin (Hb-NO) was assayed in whole blood of mice from the electron paramagnetic resonance (EPR) signal of 5-coordinate-α-Hb-NO as previously described^55^.

### Blood biochemical analyses

Plasma triglycerides, total cholesterol and non-esterified fatty acid levels were analyzed in venous blood using kits (Diasys Diagnostic and Systems, Holzheim, Germany; Randox Laboratories, Crumlin, United Kingdom). Alanine aminotransferase (ALAT) levels were measured in the serum as a marker of liver damage using the ALAT/GPT kit (DiaSys Diagnostic and Systems). Plasma concentrations of cardiovascular analytes (sE-Selectin, sICAM-1, PAI-1 Total, proMMP-9) and of cytokines (IL-6, IL-10, IL-1β, MIP1α, MCP1 and TNFα) were determined using two multiplex immunoassay kits (Milliplex kits from Merck Millipore based on Luminex® technology (Bioplex®, Bio-Rad, Belgium)). Triglycerides and cholesterol were measured in the liver tissue after extraction with chloroform–methanol according to the Folch method, as previously described^56^.

### Histological analysis

Sections of aortic arch, previously isolated from connective tissues, were frozen in embedding medium (Tissue-tek, Sakura, The Netherlands). Sections of artic arch of 5 µm were cut at 3 different levels for each sample. Haematoxylin-eosin stained sections were digitalized at a 20x magnification using a SCN400 slide scanner (Leica, Wetzlar, Germany). Frozen sections of the main liver lobe (embedding in Tissue-tek) were sliced and stained with the Oil Red O. Haematoxylin-eosin stained sections were digitalized at a 20x magnification using a SCN400 slide scanner (Leica, Wetzlar, Germany). The quantification of positive area/tissue area was determined by thresholding for the Oil Red O signal (software TissueIA, version 4.0.7).

### Real-Time quantitative PCR

Total RNA from tissues was extracted with TriPure reagent (Roche, Basel, Switzerland). cDNA was obtained by reverse transcription of 1 μg total RNA (Reverse Transcription System kit, Promega, Leiden, The Nederlands). Real-time qPCRs were performed using Mesa Fast qPCRTM (Applied Biosystems, Den Ijssel, The Netherlands and Eurogentec, Seraing, Belgium) for detection as previously described^56^. Ribosomal 7 protein L19 (RPL19) RNA was the housekeeping gene. Sequences of the primers are given in Supplementary File 1. All samples were run in duplicate and data were analyzed according to the 2^−ΔΔCT^ method.

### Western blot

Equal amount proteins from thoracic aorta and mesenteric arteries were separated by SDS/PAGE and transferred to nitrocellulose membrane, blocked with 5% non-fat dry milk or bovine serum albumin (BSA) in tris-buffered saline tween-20. The membranes were incubated overnight at 4 °C with antibodies as previously described^19^. Gels were analysed and quantified by ImageQuantTM TL instrument (GE Healthcare, Buckinghamshire, England).

### Gut microbiota analyses

Genomic DNA was extracted from the cecal content using a QIAamp DNA Stool Mini Kit (Qiagen, Hilden, Germany) according to the manufacturer’s instructions, including a bead-beating step. 16S rDNA profiling, targeting V1-V3 hypervariable region and sequenced on Illumina MiSeq were performed as described previously^57^ and detailed in Supplementary File 1. For total bacteria, *Bifidobacterium* spp., *Roseburia* spp., *Akkermansia muciniphila* and *Lactobacillus* spp. quantification by qPCR, primers are detailed in Supplementary File 1 and conditions were based on 16S rRNA gene sequence and was described earlier^21,33^. For *Alistipes* spp., method was detailed in Supplementary File 1.

### Statistical analysis

Data are presented as mean ± SEM. Within-groups variances were compared using a Bartlett’s test. If variances were significantly different between groups, values were normalized by Log-transformation before proceeding to the analysis. Dixon’s Q-test was performed to statistically reject outliers (95% confidence level). Differences between groups were assessed using one-way ANOVA, followed by the Tukey post hoc test. For cecal bacteria, we used the non-parametric Kruskal-Wallis test. Data with different superscript letters were significantly different (p ≤ 0.05) according to the post hoc ANOVA statistical analysis. Grouped analyses were assessed by two-way ANOVA. Statistical analyses were performed using GraphPad Prism version 7.04 for windows.

## Supplementary information

Supplementary File S1

Supplementary Dataset 1
