# Supplementary material for: Chitin–glucan and pomegranate polyphenols improve endothelial dysfunction
Source: Sci Rep. 2019 Oct 2;9:14150. doi: 10.1038/s41598-019-50700-4 (PMC6775069; doi:10.1038/s41598-019-50700-4)
Supplement: Supplementary file 1 — Supplementary File S1 [file 41598_2019_50700_MOESM1_ESM.docx]

**Supplementary File S1**

**Chitin–glucan and pomegranate polyphenols improve endothelial dysfunction**

Audrey M. Neyrinck, Emilie Catry, Bernard Taminiau, Patrice D. Cani, Laure B. Bindels, Georges Daube, Chantal Dessy and Nathalie M. Delzenne

Primer sequences used for quantitative PCR

|  | **Primer Forward** | **Primer Reverse** |
| --- | --- | --- |
| *Cd11c* | ACGTCAGTACAAGGAGATGTTGGA | ATCCTATTGCAGAATGCTTCTTTACC |
| *Cox2* | TGACCCCCAAGGCTCAAATAT | TGAACCCAGGTCCTCGCTTA |
| *F480* | TGACAACCAGACGGCTTGTG | CAGGCGAGGAAAAGATAGTGT |
| *Il1b* | TCGCTCAGGGTCACAAGAAA | CATCAGAGGCAAGGAGGAAAAC |
| *Lbp* | GTCCTGGGAATCTGTCCTTG | CCGGTAACCTTGCTGTTGTT |
| *Mcp1* | GCAGTTAACGCCCCACTCA | CCCAGCCTACTCATTGGGATCA |
| *Rpl19* | GAAGGTCAAAGGGAATGTGTTCA | CCTTGTCTGCCTTCAGCTTGT |
| *Tlr4* | CCCTCAGCACTCTTGATTGC | TGCTTCTGTTCCTTGACCCA |
| *Tnfa* | AGCCCCCAGTCTGTATCCTT | GGTCACTGTCCCAGCATCTT |
| Total bacteria | ACTCCTACGGGAGGCAGCAG | ATTACCGCGGCTGCTGG |
| *Alistipes* spp. | ACATAGGGGGACTGAGAG | GTTTACCCTCGTACAAAAGCA |
| *Bifidobacterium spp.* | GATTCTGGCTCAGGATGAACGC | CTGATAGGACGCGACCCCAT |
| *Lactobacillus spp.* | AGCAGTAGGGAATCTTCCA | CACCGCTACACATGGAG |
| *Roseburia spp.* | AAGCGACGATCAGTAGCCGA | TTCTTCTTCCCTGCTGATAGAG |
| *Akkermansia muciniphila* | CAGCACGTGAAGGTGGGGAC | CCTTGCGGTTGGCTTCAGAT |

*Alistipes* genus quantification by quantitative polymerase chain reaction (qPCR)

Primers and probes were designed specifically for this study and synthesized by Eurogentec (Liège, Belgium). *Alistipes* 16S reference sequences were downloaded from SILVA v1.28 database were aligned with Alistipes OTU sequences from this study using CLUSTALW (available online at http://www.ebi.ac.uk/Tools/msa/clustalo/). A minimum for three mismatches between target species and neighbors ensured the specificity for primers and probe. The standard curve is based upon 10-fold dilution of quantified target amplicon 16S rDNA of *Alistipes onderdonkii* (synthetic DNA, IDT, Leuven, Belgium). The DNA quantification is performed with a specific FAM-TAMRA probe (FAM-5’- GGT CAA TGG ACG CAA GTC TGA ACC AGC C -3'-TAMRA). Quantitative real-time PCR were performed on LC480 Lightcycler (Roche) in with Lightcycler 480 Probe master mix for a total reaction volume of 20µl. The amplification was carried out in triplicate with 40 cycles of a 95°C denaturation phase followed by a 60°C annealing phase.

16S rDNA high throughput sequencing

PCR-amplification of the V1-V3 region of the 16S rDNA and library preparation were performed with the following primers (with Illumina overhand adapters), forward

(5’-TCGTCGGCAGCGTCAGATGTGTATAAGAGACAG-3’) and reverse

(5’-GTVTVGTGGGCTCGGAGATGTGTATAAGAGACAG-3’). Each PCR product was purified with the Agencourt AMPure XP beads kit (Beckman Coulter, Pasadena, USA) and submitted to a second PCR round for indexing, using the Nextera XT index primers 1 and 2. After purification, PCR products were quantified using the Quant-IT PicoGreen (ThermoFisher Scientific, Waltham, USA) and diluted to 10 ngµL-1. A final quantification, by qPCR, of each sample in the library was performed using the KAPA SYBR® FAST qPCR Kit (KapaBiosystems, Wilmington, USA) before normalization, pooling and sequencing on a MiSeq sequencer using v3 reagents (ILLUMINA, USA).

Sequence reads processing were used as previously described using respectively MOTHUR software package v1.39.5 (1, 2) and VSEARCH algorithm (3) for alignment and clustering and chimera detection. 16S Reference alignment and taxonomical assignation were based upon the SILVA database (v1.28) of full-length 16S rDNA sequences (4).

Subsample datasets containing 10,000 read per sample were obtained and used for OTU clustering and taxonomic assignment. Good’s coverage estimator was used as a measure of sampling effort for each sample, with a mean value of 92.35%. Subsample datasets were further used to evaluate ecological indicators, richness estimation (Chao1 estimator), microbial biodiversity (reciprocal Simpson index), and the population evenness (derived from simpson index) using MOTHUR (5). Population structure, community membership were assessed with MOTHUR using distance matrice based on Bray-Curtis dissimilarity index (a measure of community structure which considers shared OTUs and their relative abundances).

Ordination analysis and 3D plots were performed with Vegan (<https://CRAN.R-project.org/package=vegan>), Vegan3d (https://CRAN.R-project.org/package=vegan3d) and rgl packages in R. Non metric dimensional scaling, based upon the Bray-Curtis dissimilarity matrix was applied to visualize the biodiversity between the groups. AMOVA test was performed to assess the diversity clustering of treatment groups with Bray-Curtis matrix using MOTHUR[5].

Statistical difference of population abundance between treatment groups were assessed with Kruskal-Wallis H tests, corrected for mutli-testing (Benjamini-Hochberg False Discovery Rate) using STAMP software (6). All the biosample raw reads have been deposited at the National Center for Biotechnology Information (NCBI) and are available under de Bioproject ID PRJNA521299.

1. Schloss PD, Westcott SL, Ryabin T, Hall JR, Hartmann M, Hollister EB, et al. Introducing mothur: open-source, platform-independent, community-supported software for describing and comparing microbial communities. ApplEnvironMicrobiol. 2009;75(23):7537-41.

2. Neyrinck AM, Etxeberria U, Taminiau B, Daube G, Van Hul M, Everard A, et al. Rhubarb extract prevents hepatic inflammation induced by acute alcohol intake, an effect related to the modulation of the gut microbiota. Mol Nutr Food Res. 2016.

3. Rognes T, Flouri T, Nichols B, Quince C, Mahe F. VSEARCH: a versatile open source tool for metagenomics. PeerJ. 2016;4:e2584.

4. Quast C, Pruesse E, Yilmaz P, Gerken J, Schweer T, Yarza P, et al. The SILVA ribosomal RNA gene database project: improved data processing and web-based tools. Nucleic Acids Res. 2013;41(Database issue):D590-6.

5. Hunter PR, Gaston MA. Numerical index of the discriminatory ability of typing systems: an application of Simpson's index of diversity. JClinMicrobiol. 1988;26(11):2465-6.

6. Parks DH, Beiko RG. Identifying biologically relevant differences between metagenomic communities. Bioinformatics. 2010;26(6):715-21.

Resting parameters and contractile profile of mesenteric arteries

Vessel diameter (**a**), basal tone (**b**) and maximal contraction (**c**, **d**) in response of KCl (50 mM) challenge in absence (**c**) or in absence (**d**) of cyclooxygenase inhibitor (indomethacin) of second- and third-order mesenteric arteries from ApoE-/- mice fed a high fat (HF) diet or a HF diet supplemented with 5% chitin-glucan (CG) or a combination of 5% CG and 0.5% pomegranate peel extracts (CG+PPE) for 8 weeks.p>0.05 (ANOVA).

Analysis of Molecular Variance results

AMOVA analysis based upon the Bray-Curtis dissimilarity matrix.

Global analysis error rate is 0.05. For the paired tests, Bonferroni correction was applied, Pair-wise error rate is 0.0083.

| **Test** | **F score** | **p-value** |
| --- | --- | --- |
| Global | 3.213 | <0.0001* |
| CT vs HF | 4.248 | 0.0002* |
| CT vs CG | 3.323 | 0.0012* |
| CT vs CG+PPE | 4.194 | 0.0002* |
| HF vs CG | 2.414 | 0.0002* |
| HF vs CG+PPE | 3.338 | 0.0003* |
| CG vs CG+PPE | 1.801 | 0.023 |
